# Self-reported fatigue in patients with rheumatoid arthritis compared to patients with cancer: results from two large-scale studies

**DOI:** 10.1007/s00296-021-04948-7

**Published:** 2021-07-16

**Authors:** Karolina Müller, Jens G. Kuipers, Joachim Weis, Irene Fischer, Tobias Pukrop, Jens U. Rüffer, Michael Koller

**Affiliations:** 1 Center for Clinical Studies, University Hospital Regensburg, Regensburg, Germany; 2 Department of Rheumatology, Red Cross Hospital Bremen, Bremen, Germany; 3 Comprehensive Cancer Center, University Clinic Center Freiburg, Freiburg, Germany; 4 Institute for Tumour-Fatigue-Research, Emskirchen, Germany; 5 Department of Oncology and Hematology, University Hospital Regensburg, Regensburg, Germany; 6 German Fatigue Society, Cologne, Germany

**Keywords:** Fatigue, Quality of life, Patient-reported outcomes, Rheumatoid arthritis, Cancer

## Abstract

Fatigue is a common symptom in patients with rheumatoid arthritis (RA) and in patients with cancer (CA). The aim was to investigate the degree of fatigue in RA patients as compared to CA patients as well as potential influencing factors on RA-related fatigue. This was a retrospective analyses of two prospective cohort studies that used the EORTC QLQ-FA12 as a common instrument to assess fatigue. The cohort of RA patients was based on a nationwide survey in Germany. The cohort of CA patients was recruited in the context of an international validation field study. Multivariable ANCOVAs compared levels of fatigue between the two cohorts, also including various subgroup analyses. Regression analyses explored influencing factors on RA patients’ fatigue. Data of *n* = 705 RA patients and of *n* = 943 CA patients were available for analyses. RA patients reported significantly higher Physical Fatigue (mean difference = 7.0, 95% CI 4.2–9.7, *p* < 0.001) and Social Sequelae (mean difference = 7.5, 95% CI 4.7–10.2, *p* < 0.001). CA patients reported higher Cognitive Fatigue (mean difference = 3.5, 95% CI 1.4–5.6, *p* = 0.001). No differences in Emotional Fatigue (*p* = 0.678) and Interference with Daily Life (*p* = 0.098) were found. In RA patients, mental health and pain were associated with fatigue (*p* values < 0.001). RA patients showed a considerable level of fatigue that is comparable to and in certain cases even higher than that of CA patients. The implementation of standardized diagnostic procedures and interventions to reduce fatigue in RA patients are recommended.

## Introduction

Fatigue is common in patients with rheumatoid arthritis (RA) as well as in patients with cancer (CA). Fatigue is defined as a condition of unusual tiredness, weakness, and exhaustion and considerably reduces the quality of life of patients [1].

In patients with RA, fatigue has an estimated prevalence of 40% to 70% and appears in all stages of the disease and during the therapeutic course [2]. The exact mechanism leading to fatigue in RA is unclear. Inflammation is thought to play an important role [3]. Fatigue is associated with the severity of pain and psychosocial factors such as depression [4]. Thus, a complex interaction of clinical factors such as disease activity, pain, and disability as well as of psychosocial and personal factors such as coping, working, beliefs, and behaviors have to be taken into account [2, 5, 6]. Since 2006, fatigue has been included as an important outcome measure in RA by the “Outcome Measures in Rheumatoid Arthritis Clinical Trials” (OMERACT) [7]. In randomized controlled trials, biologic disease-modifying anti-rheumatic drugs (DMARDs) and targeted synthetic DMARDs have been shown to have a moderate effect on improving fatigue by reducing pain and inflammation [2, 8]. Current evidence supports physical activity and cognitive behavioral interventions as the most promising approaches to reduce fatigue [9].

Numerous patient-reported outcome measures (PROM) are available to assess RA-related fatigue, including Functional Assessment of Cancer Therapy (FACT), Patient-Reported Outcomes Measurement Information System (PROMIS), and Rheumatoid Arthritis Impact of Disease (RAID) [10]. Furthermore, a core outcome set for clinical trials in this area has been proposed [11].

Fatigue is also a prominent symptom in patients with CA. Based on their systematic review and a meta-analysis of 129 studies (*N* = 71,568), Al Maqbali et al. suggested an overall prevalence of 49% of CA-related fatigue [12]. In young adulthood (median of 14 years, range 5–30), one of four CA survivors reported fatigue [13].

Fatigue in CA patients can have multiple causes, such as tumor biology, therapeutic agents, comorbidities, pain, emotional distress, anemia, sleep disturbance, nutritional deficits, or reduced functional status [14, 15]. Therefore, any decisions to alleviate fatigue have to be based on careful differential diagnostics [16]. As for RA, there is still no clear pathogenetic model to explain CA-related fatigue, albeit some hypothetical pathways have been discussed in the literature [17].

Diagnostic procedures should include a patient’s medical history as well as a structured interview [18]. In this context, validated fatigue questionnaires are crucial [19], such as the newly developed Fatigue Module of the European Organization for Research and Treatment of Cancer (EORTC QLQ-FA12) [20–22].

The goal of the present project was to investigate the degree of fatigue in RA patients as compared to CA patients using the EORTC QLQ-FA12. As this is the first study addressing this research question, and as the samples of RA and CA patients included subsamples with varying disease states, we had no clear-cut a priori hypotheses. We, therefore, proceeded with an exploratory approach. Moreover, the influence of age, sex, pain, disease activity, mental health, lifestyle, and medication on RA-related fatigue was assessed. Finally, psychometric properties of FA12 were calculated separately for both populations.

## Methods

### Study design

Two independent studies used the same fatigue assessment allowing for comparative analyses.

Data of RA patients were taken from the representative nationwide German survey TRACE (Therapie bei Rheumatoider Arthritis Correlate und Einfluss) on adherence with therapy and quality of life [23]. Patient inclusion criteria were confirmed diagnosis of RA, ongoing medical treatment, and ability to fill in a questionnaire in the German language. Patients were recruited from February 2014 to February 2015 at 67 participating centers across Germany that are specialised in the treatment of RA patients; each center was supposed to include 10 study patients or more. The TRACE study was approved by the Ethics Committee of the University of Regensburg (reference number 13-160-0275). The trial was registered with study database of the German Network of Health Services (VfD_TRACE_14_003438).

Data of CA patients were taken from an international psychometric validation study of the EORTC QLQ-FA12 measuring CA-related fatigue in eleven European and non-European countries [20–22]. Inclusion criteria were histologically confirmed cancer of any tumor site, written informed consent, and the ability to understand the language of the questionnaire. Exclusion criteria were severe psychiatric or cognitive mental conditions, age below 18 years, currently undergoing allogeneic hematological stem cell transplantation (HSCT) or neoadjuvant therapy. Patients were recruited from February 2011 to November 2014. National and local ethics approvals were obtained for the recruiting centers (reference number of leading Ethics Committee of the University of Freiburg 165/11). The study was registered with the German Clinical Trial Studies Registry (DRKS00003091).

Both studies were conducted in compliance with the Declaration of Helsinki and patients gave their written informed consent.

### Fatigue assessment

The questionnaire assesses five fatigue dimensions: Physical Fatigue, Emotional Fatigue, Cognitive Fatigue, Interference with Daily Life, and Social Sequelae. The subscales Physical, Emotional, and Cognitive Fatigue are multi-item scales, Interference with Daily Life and Social Sequelae are single items. All scores range from 0 to 100, and higher scores represent a higher symptom burden. Validation studies of the EORTC QLQ-FA12 showed excellent reliability, validity, and sensitivity to change [20, 22].

### Additional data

Sociodemographic (age and sex) information was recorded in both studies.

For CA patients, clinical information regarding tumor site, metastases, as well as the type and state of therapy were assessed and available for analyses in this study.

For RA-related fatigue, several potential influencing factors were recorded, such as disease activity, mental health, medication, current smoking, and physical activity [2, 8, 9].

The validated Disease Activity Score 28 (DAS28) was used to measure the inflammatory activity of RA [24–26]. DAS28 includes assessment of the number of swollen as well as tender joints, measurements of the erythrocyte sedimentation rate as well as of the C reactive protein, and patient self-reported assessment of global health. DAS28 values range from 0 to 10, and higher values represent higher disease activity. DAS28 < 3.2 is interpreted as remission or low disease activity, and ≥ 3.2 as moderate or high disease activity.

Short-Form-Health-Survey (SF-12, version 2) was used to measure health-related quality of life in terms of Physical and Mental Health [27, 28]. Mental Health includes emotional role functioning, mental well-being, negative affect, and social functioning. Physical Health includes general health perception, physical functioning, physical role functioning, and pain. Both scales range from 0 to 100, and higher values represent better health.

The Lifestyle Score was composed of alcohol consumption (1 = yes, seldom to daily), current smoking (1 = yes, occasional or regularly), physical exercise (1 = no), and diet (1 = primarily traditional German Food). The summary score ranged from 0 to 4, and higher scores represent a less healthy lifestyle.

### Analyses

Descriptive analyses included frequency (*n*), percentage (%), mean (*m*), and standard deviation (sd). Differences in sociodemographic variables between RA and CA patients were calculated using Chi-square test or independent *t* test, where applicable.

Differences in fatigue dimension scores between RA and CA patients were assessed using ANCOVAs (analysis of covariance). Sex and age were included as covariates in the ANCOVA models.

Two different ANCOVA models were computed. Model 1 included disease main group comparisons between RA and CA patients. Model 2 included disease subgroup analyses based on clinical considerations. RA patients were assigned to one of two subgroups according to their DAS28 score: 1. remission or low disease activity, or 2. moderate or high disease activity. CA patients were assigned to one of the following four groups: 1. receiving first-line curative therapy; 2. receiving second- or third-line palliative therapy; 3. survivors I (≥ 12 and ≤ 18 months after treatment with no evidence of cancer or recurrence); or 4. survivors II (≥ 36 and ≤ 72 months after treatment with no evidence of cancer or recurrence). Least significant differences (LSD) were used for post-hoc tests. Adjusted means including 95% confidence intervals (CI) were reported.

Multivariable linear regression analyses (enter method) were conducted to explore the influence of sex, age, pain (SF-12), Mental Health (SF-12), disease activity (DAS28, dichotomized), lifestyle, and medication on RA-related fatigue. Physical Health was not included in the model as pain is a component of the Physical Health score, which leads to a high correlation between both variables. Based on published data, pain seems to be the more relevant factor in predicting RA-related fatigue [2]. Medication was defined as: biologicals (no vs. yes), glucocorticoids (no vs. yes), and methotrexate (MTX) (no vs. yes).

Psychometric properties were assessed separately for both populations. Cronbach’s alphas for multi-item scales were computed, and values ≥ 0.70 are considered acceptable [29]. Convergent and discriminant validity were assessed by means of item-scale correlations. Acceptable indicators for convergent validity are correlation coefficients ≥ 0.40 (corrected for overlap) between an item and its own scale, and for discriminant validity, correlation coefficients < 0.40 between an item and another scale. A definite scaling error exists if an item correlated significantly less with its own scale than with another scale [30].

Normality of data was not tested, although QoL data occasionally may be skewed. Nevertheless, by default QoL data are presented as means ± standard deviations [31]. Moreover, Gaussian models are remarkably robust to violations of normality assumption [32].

The level of significance was set at *p* ≤ 0.05 for all tests. Since this was an exploratory study, no adjustments for multiple testing were made [33]. All analyses were performed using the software package SPSS (Version 26, SPSS Inc, Chicago, Illinois).

## Results

### Sample

In total, 708 RA patients had been included in the original TRACE study [23]. Three patients were excluded from the present analyses due to missing fatigue assessment, resulting in 705 RA patients. RA patients characteristics are presented in Table 1, mean age was 59.5 years (sd = 12), and the majority of patients were women (73%).

**Table 1:** Characteristics of patients with rheumatoid arthritis (*N* = 705)

*Age* in years; mean ± SD (range)	59.5 ± 12.0 (20–90, *n* = 687)
*Sex*, No. (%)	
Male	192 (27.2%)
Female	512 (72.7%)
Missing	1 (0.1%)
*Alcohol*; No. (%)	
Yes, daily	23 (3.3%)
Yes, several times a week	48 (6.8%)
Yes, once a week	113 (16.0%)
Yes, once a month	59 (8.4%)
Yes, seldom	207 (29.4%)
No	252 (35.7%)
Missing	3 (0.4%)
*Smoking*; No. (%)	
Yes, regularly	90 (12.8%)
Yes, occasionally	47 (6.7%)
No, ex-smoker	88 (12.5%)
No	475 (67.4%)
Missing	5 (0.7%)
*Physical exercise*; No. (%)	
No	227 (32.2%)
Yes, once a week for 45 min	183 (26.0%)
Yes, twice a week for 45 min	127 (18.0%)
Yes, three times a week for 45 min	77 (10.9%)
More often	79 (11.2%)
Missing	12 (1.7%)
*Food*; No. (%)	
Primarily traditional German food	444 (63.0%)
Primarily Mediterranean food	172 (24.4%)
Primarily vegetarian food	41 (5.8%)
Missing	48 (6.8%)
*Lifestyle Score*^**a**^; Mean ± SD (range)	1.8 ± 1.0 (0–4, *n* = 641)
*DAS28*; mean ± SD (range)	2.8 ± 1.2 (0–7, *n* = 679)
*DAS28*; No. (%)	
Remission or low disease activity	456 (64.7%)
Moderate or high disease activity	223 (31.6%)
Missing	26 (3.7%)
*Mental Health* (SF-12); Mean ± SD (range)	48.2 ± 11.4 (17.3–69.8, *n* = 603)
*Physical Health* (SF-12); Mean ± SD (range)	41.7 ± 10.6 (15.1–63.0, *n* = 603)
*Pain* (SF-12); No. (%)	
Not at all	202 (28.7%)
A little	206 (29.2%)
Moderately	165 (23.4%)
Quite a bit	90 (12.8%)
Very	24 (3.4%)
Missing	18 (2.6%)
*Biologicals*; No. (%)	
No	441 (62.6%)
Yes	264 (37.4%)
*Glucocorticoids*; No. (%)	
No	307 (43.5%)
Yes	398 (56.5%)
*Methotrexate*; No. (%)	
No	313 (44.4%)
Yes	392 (55.6%)
*Length of patient-physician relationship*; No. (%)	
Up to 6 months	64 (9.1%)
Up to 1 year	48 (6.8%)
Up to 2 years	111 (15.7%)
Up to 5 years	179 (25.4%)
Longer	300 (42.6%)
Missing	3 (0.4%)

^a^Data from four single lifestyle factors alcohol, smoking, physical exercise, and food were combined to a score ranging from 0 to 4, whereas a higher score represents a less healthy lifestyle

In total, 946 CA patients with a broad range of tumor sites were included in the original study [20–22], but three patients were excluded due to missing fatigue assessment, resulting in 943 CA patients. Mean age was 58.3 years (sd = 13, *n* = 942), and the majority of patients were women (*n* = 510, 54%). Tumor sites were as follows: breast *n* = 225 (24%), head/neck *n* = 213 (23%), lung *n* = 105 (11%), colorectal *n* = 90 (10%), gynecological *n* = 61 (6%), prostate *n* = 61 (6%), hematological *n* = 49 (5%), and other *n* = 145 (15%). 25% of patients had metastases (*n* = 232), whereas 71% (*n* = 668) had no metastases, and for 5% (*n* = 43) information was missing.

The two patient groups differed significantly in sex (*p* < 0.001) but not in age (*p* = 0.057).

### Differences in fatigue

ANCOVAs were used to compare fatigue dimensions between disease groups adjusted for sex and age. Two definitions of disease groups were used. Model 1 included two main groups: RA patients (*n* = 705) vs. CA patients (*n* = 943). To account for clinical status, model 2 included six subgroups: 1. RA patients in remission or with low disease activity (*n* = 456), 2. RA patients with moderate or high disease activity (*n* = 223), 3. CA patients receiving first-line therapy (*n* = 309), 4. CA patients receiving second- or third-line therapy (*n* = 222), 5. CA survivors I (≥ 12 and ≤ 18 months after treatment with no evidence of cancer or recurrence, *n* = 212), and 6. CA survivors II (≥ 36 and ≤ 72 months after treatment with no evidence of cancer or recurrence, *n* = 198). For 26 RA and for 2 CA patients clinical information (DAS28/therapy) was missing, thus they were excluded from model 2.

#### Model 1

RA and CA patients significantly differed in Physical Fatigue, Cognitive Fatigue, and Social Sequelae (Table 2). RA patients reported significantly higher Physical Fatigue (mean difference = 7.0, 95% CI 4.2–9.7, *p* < 0.001) and Social Sequelae (mean difference = 7.5, 95% CI 4.7–10.2, *p* < 0.001) than CA patients. CA patients reported higher Cognitive Fatigue impairment than RA patients (mean difference = 3.5, 95% CI 1.4–5.6, *p* = 0.001). No differences in Emotional Fatigue (*p* = 0.678) and Interference with Daily Life (*p* = 0.098) were found between the two patient groups.

**Table 2:** Differences in EORTC QLQ-FA12 dimensions between patients with rheumatoid arthritis and cancer

Disease	*n*	Physical fatigue			Emotional fatigue			Cognitive fatigue			Interference with daily life			Social sequelae		
		*m*	*95% CI*		*m*	*95% CI*		*m*	*95% CI*		*m*	*95% CI*		*m*	*95% CI*	
ANCOVA model 1 (*N* = 1648)																
Rheumatoid arthritis patients	705	43.0	40.8	45.2	25.3	23.1	27.5	10.0	8.4	11.7	34.2	31.6	36.7	19.4	17.2	21.5
Cancer patients	943	36.0	34.3	37.8	25.9	24.1	27.7	13.5	12.2	14.8	36.9	34.9	39.0	11.8	10.1	13.6
*p*		** < 0.001**			0.678			**0.001**			0.098			** < 0.001**		
ANCOVA model 2 (*N* = 1620)																
Rheumatoid arthritis patients—remission/low disease activity	456	39.5	36.9	42.1	21.3	18.7	23.9	8.9	6.9	10.9	30.6	27.5	33.6	16.2	13.6	18.8
Rheumatoid arthritis patients—moderate/high disease activity	223	50.4	46.7	54.1	32.7	29.0	36.5	12.0	9.2	14.9	41.4	37.0	45.8	25.7	22.0	29.4
Cancer patients—first-line therapy	309	34.5	31.5	37.5	25.8	22.8	28.9	11.5	9.2	13.8	34.5	31.0	38.0	9.7	6.7	12.6
Cancer patients—second- or third-line therapy	222	45.8	42.3	49.4	37.0	33.4	40.6	17.0	14.2	19.7	51.6	47.5	55.8	11.0	7.5	14.6
Cancer patients—survivors I (12–18 month off treatment)	212	33.4	29.8	37.0	20.9	17.2	24.6	13.6	10.8	16.4	31.8	27.5	36.1	14.7	11.1	18.3
Cancer patients—survivors II (36–72 month off treatment)	198	30.4	26.6	34.1	19.2	15.4	23.0	12.7	9.8	15.6	30.2	25.7	34.6	13.2	9.5	16.9
*p*		** < 0.001**			** < 0.001**			** < 0.001**			** < 0.001**			** < 0.001**		

Higher EORTC QLQ-FA12 scores represent higher impairment in fatigue (ranges from 0 to 100). Means were adjusted for sex and age. Clinical information was missing for 26 patients with rheumatoid arthritis and 2 cancer patients; thus, these patients were excluded from the ANCOVA model 2 analysis. The actual number of patients was occasionally smaller because of missing values

Bold values denote statistical significance at the p ≤ 0.05 level

#### Model 2

Table 2 present differences in fatigue dimensions between disease subgroups. RA patients with moderate/high disease activity reported higher Physical (*p* < 0.001), Emotional (*p* < 0.001), and Cognitive (*p* = 0.069) Fatigue as well as Interference with Daily Life (*p* < 0.001) and Social Sequelae (*p* < 0.001) than RA patients in remission/with low disease activity.

Within subgroups of CA patients, patients receiving second- or third-line therapy reported highest interference with fatigue dimensions compared to the other CA patient subgroups (*p* values < 0.05), except for Social Sequelae. CA survivors I and II did not significantly differ in any fatigue dimension (*p* values > 0.05). Patients receiving first-line therapy reported higher impairment of Emotional Fatigue than CA survivors I and II (*p* values < 0.05). CA survivors I reported higher impairment of Social Sequelae than patients receiving curative therapy (*p* = 0.035).

RA patients in remission/with low disease activity reported impairment in Emotional Fatigue and Social Sequelae comparable to those of CA survivors I and II, but significantly lower Emotional Fatigue impairment and higher Social Sequelae impairment than CA patients in both therapy groups (*p* values < 0.05). Moreover, Physical Fatigue was significantly higher in RA patients in remission/with low disease activity than in CA patients receiving first-line therapy as well as in CA survivors I and II (*p* values < 0.05). CA patients receiving second- or third-line therapy reported higher Physical Fatigue as well as Interference with Daily Life than RA patients in remission/with low disease activity (*p* values < 0.01). Cognitive Fatigue values were significantly lower in RA patients in remission/with low disease activity than in CA patients receiving second- or third-line therapy as well as in CA survivors I and II (*p* values < 0.05).

RA patients with moderate/high disease activity reported comparable Physical and Emotional Fatigue values (*p* values < 0.05), and lower impairment in Cognitive Fatigue (*p* = 0.014) as well as Interference with Daily Life (*p* = 0.001) than CA patients receiving second- or third-line therapy. Social Sequelae was significantly higher in RA patients with moderate/high disease activity than in all CA subgroups (*p* values < 0.001). Moreover, RA patients with moderate/high disease activity reported higher impairment in Physical Fatigue, Emotional Fatigue, and Interference with Daily Life than CA patients receiving first-line therapy and CA survivors I and II (*p* values < 0.05). Cognitive Fatigue was comparable in RA patients with moderate/high disease activity and CA patients receiving first-line therapy as well as CA survivors I and II (*p* values > 0.05).

### Predictors of RA-related fatigue

Multivariable regression analyses showed that Mental Health (SF-12) was constantly associated with all five fatigue dimensions (Table 3). With increasing Mental Health scores (better health), the fatigue scores decreased (less symptom burden) (*p* values < 0.001). Pain (SF-12) was significantly associated with four out of five fatigue dimensions (Physical, Emotional as well as Cognitive Fatigue, and Interference with Daily Life). With increasing pain, the fatigue scores increased (more symptom burden) (*p* values < 0.001, Table 3). Patients taking glucocorticoids reported on average 4.6 (95% CI 1.6–7.7) higher Emotional Fatigue than patients who did not (*p* = 0.003). With increasing lifestyle scores (less healthy lifestyle), the Interference with Daily life decreased (*p* = 0.040). With increasing age, the Social Sequalae decreased (*p* = 0.012). No further, significant associations were found (Table 3).

**Table 3:** Predictors of rheumatoid arthritis related fatigue

	*B*	95% CI		*p*
Physical fatigue (*N* = 521)				
Age	− 0.12	− 0.25	0.01	0.080
Sex	1.77	− 1.88	5.42	0.341
DAS28	− 1.03	− 4.55	2.49	0.566
Pain (SF-12)	9.42	7.78	11.06	** < 0.001**
Mental health (SF-12)	− 1.27	− 1.42	− 1.12	** < 0.001**
Lifestyle	0.06	− 1.52	1.63	0.945
Biologicals	2.64	− 0.58	5.85	0.108
Glucocorticoids	2.02	− 1.16	5.21	0.212
Methotrexate	− 1.48	− 4.66	1.69	0.359
Emotional fatigue (*N* = 519)				
Age	− 0.05	− 0.18	0.07	0.416
Sex	− 0.45	− 3.91	3.01	0.800
DAS28	1.19	− 2.16	4.54	0.486
Pain (SF-12)	5.93	4.38	7.49	** < 0.001**
Mental health (SF-12)	− 1.56	− 1.70	− 1.41	** < 0.001**
Lifestyle	0.37	− 1.12	1.86	0.630
Biologicals	2.06	− 1.00	5.12	0.187
Glucocorticoids	4.64	1.61	7.67	0**.003**
Methotrexate	0.13	− 2.89	3.15	0.933
Cognitive fatigue (*N* = 511)				
Age	− 0.05	− 0.15	0.05	0.320
Sex	− 1.42	− 4.16	1.32	0.310
DAS28	− 0.92	− 3.59	1.75	0.498
Pain (SF-12)	2.44	1.20	3.68	** < 0.001**
Mental Health (SF-12)	− 0.69	− 0.81	− .58	** < 0.001**
Lifestyle	− 0.79	− 1.98	0.40	0.193
Biologicals	0.20	− 2.24	2.64	0.870
Glucocorticoids	− 0.03	− 2.45	2.38	0.979
Methotrexate	1.97	− 0.44	4.37	0.109
Interference with daily life (*N* = 509)				
Age	− 0.18	− 0.37	0.00	0.056
Sex	− 0.47	− 5.57	4.64	0.858
DAS28	− 0.19	− 5.15	4.76	0.939
Pain (SF-12)	6.74	4.45	9.04	** < 0.001**
Mental health (SF-12)	− 1.34	− 1.55	− 1.13	** < 0.001**
Lifestyle	− 2.32	− 4.54	− 0.10	**0.040**
Biologicals	3.71	− 0.81	8.23	0.107
Glucocorticoids	0.41	− 4.08	4.90	0.858
Methotrexate	− 1.43	− 5.89	3.02	0.528
Social sequelae (*N* = 496)				
Age	− 0.23	− 0.42	− 0.05	**0.012**
Sex	− 4.00	− 8.96	0.95	0.113
DAS28	1.46	− 3.36	6.29	0.552
Pain (SF-12)	1.97	− 0.26	4.20	0.083
Mental health (SF-12)	− 1.39	− 1.60	− 1.19	** < 0.001**
Lifestyle	0.57	− 1.57	2.72	0.599
Biologicals	2.50	− 1.88	6.89	0.262
Glucocorticoids	3.92	− 0.43	8.28	0.077
Methotrexate	1.40	− 2.92	5.72	0.525

Five multivariable regression analyses were conducted; requirements were checked and outliers defined by standardized residuals > 3 excluded from analyses. DAS28 was entered dichotomously: reference category was remission or low disease activity. Reference categories for the other dichotomous variables were: sex = male, medication defined as biologicals, glucocorticoids, methotrexate = no

Bold values denote statistical significance at the p ≤ 0.05 level

### Psychometric properties

We obtained acceptable to excellent internal consistencies of multi-item scales of the EORTC QLQ-FA12, which were practically identical for RA (Cronbach’s alphas = 0.74–0.92) and for CA patients (Cronbach’s alphas = 0.81–0.90) (Table 4). Moreover, the proposed scale structure of the multi-item scales were confirmed in both samples (Table 4).

**Table 4:** Reliability, convergent and discriminant validity of EORTC QLQ-FA12 of patients with rheumatoid arthritis and cancer

Item Number	Physical Fatigue		Emotional Fatigue		Cognitive Fatigue		Interference with Daily Life		Social Sequelae	
	*RA*	CA	*RA*	CA	*RA*	CA	*RA*	CA	*RA*	CA
1	*0.82*	*0.82*	0.69	0.50	0.45	0.44	0.66	0.69	0.51	0.34
2	*0.85*	*0.81*	0.69	0.53	0.46	0.47	0.67	0.71	0.49	0.35
3	*0.79*	*0.77*	0.70	0.47	0.45	0.47	0.63	0.68	0.45	0.32
4	*0.72*	*0.68*	0.62	0.45	0.45	0.44	0.76	0.61	0.51	0.34
5	*0.79*	*0.73*	0.74	0.56	0.53	0.50	0.68	0.62	0.52	0.34
6	0.76	0.54	*0.81*	*0.72*	0.61	0.45	0.60	0.48	0.53	0.34
7	0.70	0.51	*0.83*	*0.74*	0.61	0.41	0.55	0.49	0.48	0.28
8	0.73	0.49	*0.82*	*0.71*	0.59	0.44	0.57	0.44	0.54	0.38
9	0.59	0.55	0.67	0.47	*0.64*	*0.69*	0.53	0.43	0.48	0.40
10	0.35	0.45	0.49	0.44	*0.64*	*0.69*	0.36	0.41	0.37	0.38
11	0.78	0.78	0.62	0.54	0.50	0.46	–	–	0.63	0.37
12	0.57	0.40	0.55	0.38	0.48	0.42	0.63	0.37	–	–
Cronbach’s alpha	0.92	0.90	0.91	0.85	0.74	0.81	–	–	–	–

*RA* rheumatoid arthritis patients (*N* = 705), *CA* cancer patients (*N* = 943). The actual number of patients was occasionally smaller because of missing values

Cronbach’s alpha values for multi-item scales were > 0.70 in both populations and are considered acceptable indicators for internal consistency [29]. Correlation coefficients supported convergent validity (item-own-scale correlations coefficients ≥ 0.40; corrected for overlap, italic values) and discriminant validity (item-other-scale correlations coefficients < 0.40 or no definite scaling error) in both populations [30]

## Discussion

A major finding of the present study was that Physical Fatigue was higher in RA patients than in CA patients. The highest level of Physical Fatigue was found in RA patients with high disease activity, which was even slightly higher than that of CA patients with second- or third-line therapy. RA patients also reported a higher degree of Social Sequelae, and again the highest means were found in RA patients with high disease activity.

One reason for the relatively lower fatigue values of CA patients than those of RA patients may be awareness of the topic and research activity in the CA community. According to Maqbali et al. [12], who compared studies on the prevalence of CA-related fatigue published from 1996 to 2000 with studies published from 2016 to 2020, prevalence of CA-related fatigue has decreased over the years, probably due to the fact that several clinical guidelines have been published on the assessment and management of CA-related fatigue [15, 34, 35]. Most oncological and related guidelines refer to fatigue [36–41]. In addition, evidence-based Self-Help-Management-Programs for CA patients have become available [42–44].

Another reason for CA/RA differences in fatigue may be due to pathophysiology. Fatigue was higher in RA patients with moderate/high disease activity than in RA patients with remission/low disease activity. For four out of five fatigue dimensions, the mean difference was around 10 score points or higher (Physical, Emotional, Interference with Daily Life, Social Sequalae).

As fatigue is a complex and multifactorial process in all disease groups, possible differences in fatigue-influencing factors (e.g., different ongoing treatments, somatic comorbidities, pain, depression, anxiety, decreased functional status) may exist, thus affecting the results.

A slightly different picture emerged regarding Cognitive Fatigue. Here, on average, CA patients scored higher than RA patients, with CA patients undergoing second- or third-line care yielding the highest scores. One reason for this may be that CA patients frequently complain about neuropsychological deficits due to adjuvant therapy. However, it is difficult to differentiate between Cognitive Fatigue and CA-related cognitive impairment (chemotherapy-related cognitive impairment [CRCI], Chemobrain). For this purpose, additional neuropsychological assessment would have been necessary, which was not focus of this study. CRCI can occur during or after chemotherapy and represents a concern for many patients with CA [45], but possibly not for patients with RA because of the different treatment, although some drugs like methotrexate are used for RA and CA.

CA patients undergoing second- or third-line therapy also had high scores regarding Interference with Daily Life and Emotional Fatigue, followed by RA patients with high disease activity. On average, RA and CA patients did not differ in Interference with Daily Life and Emotional Fatigue.

Given the high overall fatigue level in RA patients, we conducted regression analyses to explore potential influencing variables. We found that Mental Health and Pain were associated with all or most fatigue dimensions. In our study, medication did not play a role regarding fatigue, except for glucocorticoids, which led to higher Emotional Fatigue than in patients who did not take glucocorticoids. This correlation may be explained by the fact that glucocorticoids influence psychiatric and cognitive functions and cause sleep disturbances [46]. When entered into a multivariable model, the effect of disease activity (DAS28) vanished. This observation is in line with previous studies that identified pain and psychosocial factors to be more important for the development of fatigue than disease activity [4, 47]. Thus, the differences in fatigue found between our RA patients with moderate/high vs. low disease may be due to underlying factors that are closely connected with disease activity.

To our knowledge, this is the first study that directly compared fatigue levels in RA and CA patients. A qualitative meta-analysis explored fatigue sensations in different non-CA patient populations, such as RA, chronic kidney disease, chronic obstructive pulmonary disease, heart failure, and multiple sclerosis [1].This analysis found that fatigue was common across these diseases and diminished patients’ quality of life. The present study adds important information regarding quantitative differences and showed that RA patients, on average, were worse off than CA patients in two out of five fatigue dimensions. In some sense, the present project follows the rationale of the famous Medical Outcome Study (MOS), which investigated variations in patient outcomes in different disease groups such as diabetes, hypertension, and coronary heart disease [48].

The present study also showed that the EORTC QLQ-FA12, developed to assess fatigue in CA patients, is also a suitable questionnaire with appropriate psychometric properties for RA patients. Internal consistencies as well as convergent and discriminant validity of the multi-item scales were comparably high in both populations. Moreover, the EORTC QLQ-FA12 was able to distinguish between groups of different disease burden.

One limiting factor of the present analysis is that RA patients were entirely German in contrast to CA patients who came from eleven European and Non-European countries. Therefore, the generalizability of the psychometric results is limited. However, the psychometrics were similar to the ones found in the international sample of CA patients. Therefore, we interpret our results with a high degree of confidence. A strength of this project was the high number of patients, which allowed to reliably investigate subgroup comparisons.

Another limiting factor of this study was that data were based on two studies that were originally designed for different aims. The TRACE study investigated adherence with therapy and quality of life of patients with RA, and the EORTC study was conducted as an international validation study of the QLQ-FA12. Therefore, numerous clinical variables that would have been of interest in the present analyses (e.g., anemia, specifics of cancer medication) were not available. Nevertheless, these potential “confounding” factors can be thought of as characteristics of the study populations under investigation. This was exactly the purpose of this project: approaching two patient groups (RA and CA) with all their known and unknown characteristics and exploring their differences with regard to quality of life.

## Conclusion

The fatigue level of RA patients should be regarded as alarming, because it was comparable to and sometimes even higher than that of CA patients. There is an urgent need to generate more knowledge on RA-related fatigue. To start with, the implementation of current standardized diagnostic procedures and interventions to reduce fatigue in RA patients are recommended. This study also showed that the EORTC QLQ-FA12, developed to measure fatigue in CA patients, is also suitable and reliable for RA patients.

## Data Availability

The data sets used and/or analyzed during the current study are available from the corresponding author on reasonable request.
